# The Effects of Combination of Mimic miR-155-5p and Antagonist miR-324-5p Encapsulated Chitosan in Ovarian Cancer SKOV3

**DOI:** 10.31557/APJCP.2020.21.9.2603

**Published:** 2020-09

**Authors:** Rahma B Suardi, Ysrafil Ysrafil, Salsabila L Sesotyosari, Ronny Martien, Tirta Wardana, Indwiani Astuti, Sofia M Haryana

**Affiliations:** 1 *Study Program of Biotechnology, Graduate School, Universitas Gadjah Mada, Yogyakarta, Indonesia. *; 2 *Faculty of Medicine, Public Health, and Nursing, Universitas Gadjah Mada, Yogyakarta, Indonesia.*; 3 *Faculty of Pharmacy, Universitas Gadjah Mada, Yogyakarta, Indonesia. *; 4 *Universitas Jenderal Soedirman, Central Java, Indonesia. *

**Keywords:** Mimic miR, 155-5p, antagonist miR-324-5p, chitosan, cell line SKOV3

## Abstract

**Objective:**

Ovarian cancer is a malignant tumor that attacks reproductive organs of women. MicroRNA is known to have an involvement in the prognosis of ovarian cancer. One of them is miR-155-5p which is down regulated and miR-324-5p which is up regulated. Chitosan is used as microRNA delivery system. The aims of this study is to find out the effects of combination microRNA encapsulated chitosan in cell line SKOV3.

**Methods:**

Cell line SKOV3 obtained from Stem Cell and Cancer Institute (Kalbe). Mimic miR-155-5p and Antagonist miR-324-5p formulated with chitosan. Total RNA was extracted from nine samples (three as control and six as treatment), and prepared for cDNA synthesis. Expression of RNA and mRNA target was measured using q-PCR Biorad CFX96 C.100 and Gen Ex 7 software. Statistics analysis was measured using SPSS 16.0.

**Results:**

The administration of combination microRNA encapsulated with chitosan affect the expression of *miR-155-5p* and *miR-324-5p* endogen (P<0.05). The expression of mRNA target HIF1α and GLI1 was down regulated after treatment. The correlation between expression of microRNA and mRNA target was strongly (P<0.05).

**Conclusion:**

This study successfully presented effects of combination of mimic miR-155-5p and antagonist miR-324-5p encapsulated chitosan which be considered as a potential therapy targets for ovarium cancer.

## Introduction

Ovarian cancer is one of the three main types of malignant tumors among all female gynecological tumors. Ovarian cancer exhibits no specific clinical symptoms in the early stage, resulting in the majority of patients coming in a state of cancer cells metastasis at the time of diagnosis (Li et al., 2016). The latest report indicate that the five-year survival rate of ovarian cancer is 42.9%. However, more than 80% of patients with advanced ovarian cancer will relapse with a very poor prognosis. The extremely poor prognosis of ovarian cancer is known to be related to abnormal microRNA expression (Chen et al., 2019). 

Micro RNAs (miRNAs) were discovered to be the endogenous non-coding small RNA molecules consisting of around 22 nucleotides. After transcription, the miRNAs regulate translation and expression of the mRNA target genes (Ferretti et al., 2008). Chasanah et al., (2016) found that expression of *hsa-miR-155-5p* had decreased in the blood plasma of ovarian cancer. Hsa-miR-155-5p is a group of miRNA suppressor tumors that can bind and supress *HIF1α* expression. HIF1α is the mRNA target of miR-155-5p which functions as defense mechanism cancer cells in Hypoxia. Sumadi et al., (2018) also found that expression of *miR-324-5p* increased in cell line SKOV3. *Mir-324-5p* is oncogenic and suppress tumor suppressor gene expression. Based on in silico research by Nurasih et al., (2018) miR-324-5p targeting GLI1 through Hedgehog signaling pathway. 

Miroshnichenko et al., (2019) demonstrated the success of miRNA-based therapy combined with chemotherapy drugs to inhibit tumor development. Recent studies have shown a considerable increase in the efficacy of anticancer therapy as a synergistic effect of the therapy being applied simultaneously. MicroRNA derived from the same or different miRNA family, a mixture of anti-miRNA oligonucleotides and cytostatic drugs, and a combination of synthetic miRNA, have a complex effect compared with single miRNA therapy.

The success of administration miRNA into cancer cells is determined by dependence of specific, efficient and safe delivery. Chemical modification of miRNA synthetic could reduce miRNA ability. The development of the miRNA delivery system is focused use of nanoparticles transfection vectors. Nanoparticles are colloidal particles that range 1-10 nm, and are formulated using biodegradable polymers. One of the cationic polymers that have been utilized as delivery system using nanoparticles is chitosan. 

Chitosan is a biodegradable polysaccharide composed of two D-glucosamine and N-acetyl-D-glucosamine subunits which are bound together with (1,4) glycosidic bonds. The cationic properties of chitosan in acidic conditions enable the formation of complexes with negatively charged RNA (Winarti et al., 2011; Santos-Carballal et al., 2015). Deng et al (2014) using doxorubicin and miR-34a are encapsulated with acid hyaluronic-chitosan nanoparticles are proven to provide a synergistic effect on tumor suppression. This study was focused on the in vitro evaluation of the plausible application of combination miR-155-5p and miR-324-5p with chitosan nanoparticles in SKOV3 cell.

## Materials

*Materials*

Cell lines SKOV3 was purchased from Stem Cell and Cancer Institute Kalbe. Mimic miR-155-5p and antogonist miR-324-5p were purchased from Integrated DNA Technologies, USA. Chitosan medium moleculer weight was purchased from Sigma-Aldrich®. Primer mix miR-155-5p dan miR-324-5p (Intergrated DNA Technology, USA), Primer mRNA HIF1α, GLI1 dan β-actin (Intergrated DNA Technology, USA), miRCURY RNA Isolation Kit Cell and Plants (Cat. No.300110, Exiqon), Universal cDNA synthesis kit II 8-64 rxns (Cat. No.203301, Exiqon), ExiLent SYBR Green master mix, 2.5 ml (Cat. No.203402, Exiqon), SensiFAST™ SYBR® (Cat. No. BIO-98020, Bioline). 

Cell culture and chitosan microRNA formulation

The SKOV3 cell was cultured in DMEM culture medium containing 10% fetal bovine serum (FBS) and placed in 5% CO_2_ incubator chamber at 37^o^C. Experimental groups were divided into control and combination miRNA transfected groups. Chitosan nanoparticles were prepared following the ionic gelation method using tripholyphospate (TPP) as cross linker. The chitosan-TPP transfection reagent, miR-155-5p mimic and antagonist miR-324-5p were mixed in a ratio 1:1 by volume, incubated in serum-free medium for 20 minutes. 

*Characterization of nanoparticles chitosan*

Nanoparticles microRNA complex determined using electrophoresis according to protocol. The morphology of nanoparticles was evaluated using a transmission electron microscope (JEM-1400 JEOL). Samples were analyzed at 120 kV. A drop of the sample was deposited on a copper screen coated with carbon. The sample was dried and then contrasted with uranyl acetate for 2 min and then washed with distilled water. 

*In vitro cytotoxic activity of nanoparticles*

The cultured cells were plated in 96-well culture dishes (6x10^3^ cells), incubated for 24h in a humidified atmosphere of 5% CO_2_ and then treated with different concentrations of empty or combination miRNA loaded nanoparticles, incubation for 24h. A quantity of 100 µl MTT was added in each well at four hours before testing. Formazan crystal dissolved in 100 µl SDS were added to each well. After overnight incubation, the absorbency was measured by ELISA reader.

*Oligonucleotide transfected into SKOV3 cell in vitro*

SKOV3 cell of each group with concentration of 7x10^5^/ml were seed in six well plates and incubated at 37^o^C, in 5% CO_2_ incubator for 24h. Then added chitosan nanoparticles combination microRNA to the wells and cultured. After 24 hours, RNA was isolated. 

*RNA isolation and quantification*

Total RNA extraction was isolated using miRCURY RNA Isolation Kit Cell and Plants, in compliance with the manufacturer’s intructions. The total RNA from each samples were quantified using nanodrop to determine the RNA purity. The cDNA libraries were generated using Universal cDNA synthesis kit II 8-64 rxns. The quantification of expression *miR-155-5p* and miR-324-5p using ExiLent SYBR Green master mix, 2.5 ml. The quantification of expression *mRNA HIF1α* and GLI1 using SensiFAST™ SYBR^®^. The quantification of expression of *miRNA* and *mRNA* using quantitative PCR Biorad CFX96 C.100 (Bio-Rad^®^). The expression was analyzed using Biorad CFX Manager™ Software and GenEx 7.

*Statistical analysis*

One-way ANOVA was used for the statistical analysis of the various experiments. A posteriori Bonferroni t-test was carried out to check the ANOVA test. A p value of <0.05 was considered statiscally significant. 

## Results

*Characterization of nanoparticles chitosan*

**Figure 1:**
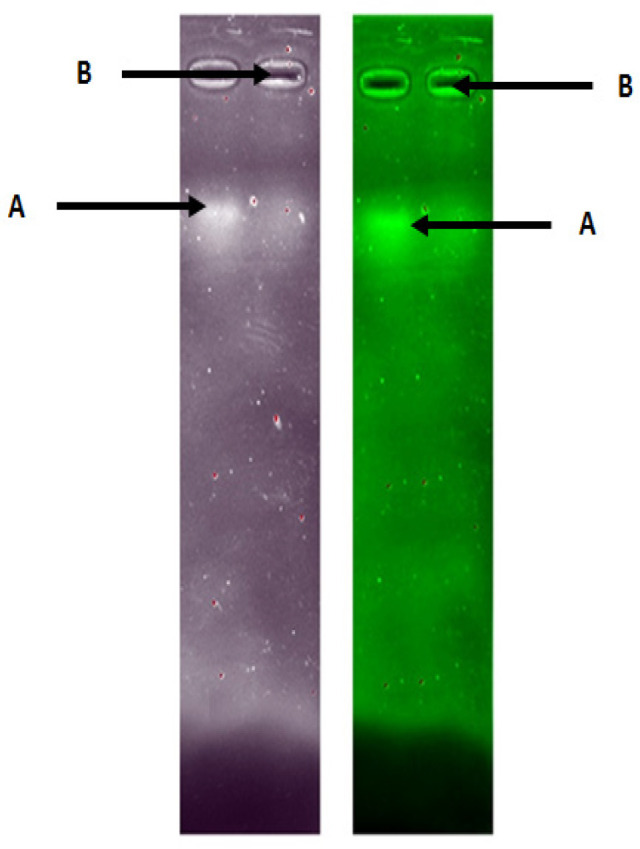
Chitosan-microRNA Nanoparticles were Run at 50 V for 20 Minutes. Note: (A) combination of naked microRNA, (B) combination of microRNA encapsulated chitosan

**Figure 2:**
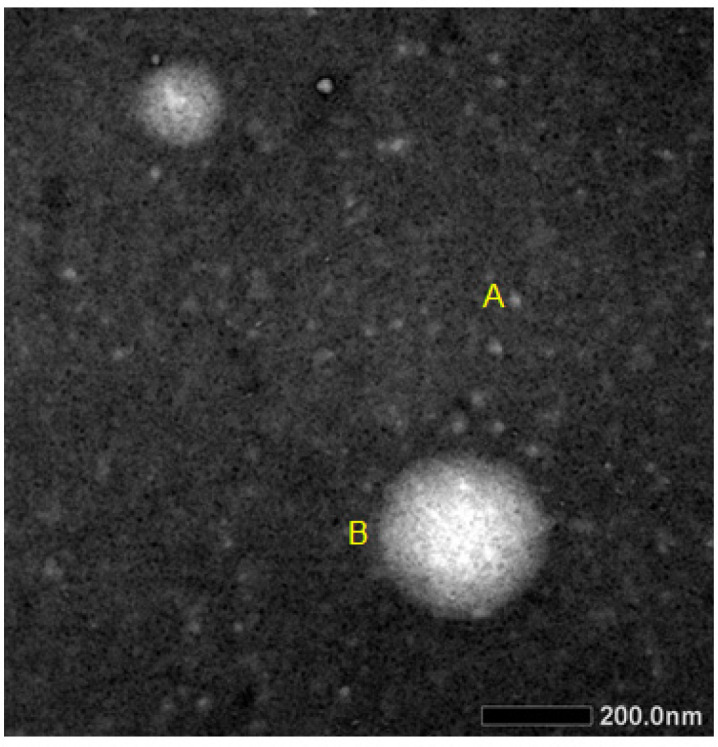
The Morphology of the Nanoparticles was Evaluated Using TEM. Samples were analyzed at 120 kV. TEM micrographs of empty nanoparticles/microRNA (A) and of nanoparticles/microRNA (B).

The ability of nanoparticles to interact and retain miRNA and the effective encapsulation were qualitatively investigated by agarose gel electrophoresis. Electrophoresis results showed that the microRNA combination complex without chitosan migrated from the well, while the chitosan-combination microRNA complex remained in the well (Figure 1). TEM analysis results the encapsulation of combination miR-155-5p and miR-324-5p with chitosan did not modify the morphology of the nanoplexes, which were characterized by a smooth spherical shape (Figure 2). 

*In vitro cytotoxic activity of nanoparticles*

**Figure 3:**
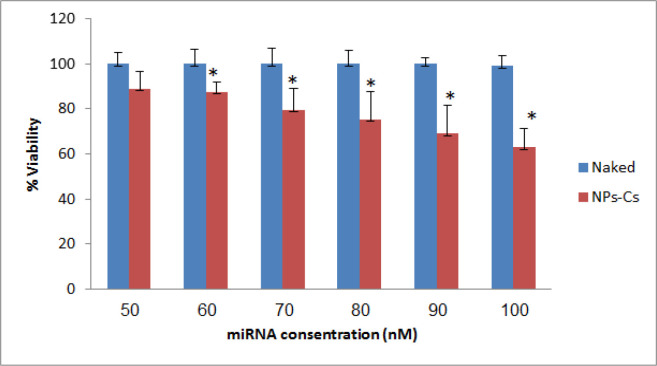
Percentage of inhibition of cell viability treated with chitosan-miRNA combination nanoparticles compared to combination treatment with miRNA without chitosan. Data are reported as the average mean ± standart deviation. *P<0.05. Naked: miRNA combination without chitosan; NPs-Cs: chitosan nanoparticles-miRNA combination

The in vitro antitumor effect of combination miRNA-loaded chitosan in terms of dose-response was investigated by using the MTT-test on SKOV3. The extremely low toxicity of empty chitosan towards SKOV3 cells was demonstrated, which means that any anti-proliferative effect that occurs has to be entrapped miRNA. Then it was observed that a significant decrease in cell viability was brought about by the chitosan nanoparticles at the highest combination miRNA (100 nm) after 24 h incubation. In particular, at a combination miRNA concentration of 100 nm, the nanoplexes induced a decrease in cell viability of 40% on SKOV3 cells (Figure 3). There was significant difference between the transfected chitosan-miRNA groups compared to naked miRNA (P<0.05). 

*MiR-155-5p expression and its correlation with HIF1α*

**Figure 4:**
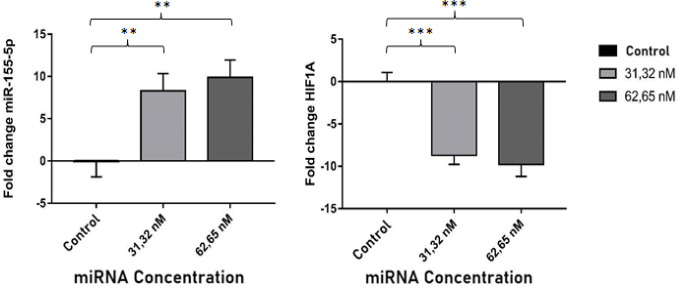
Fold Change of Endogenous miRNA-155-5p and HIF1α mRNA Expression after Tranfected NPs-CS Combination of miRNA 31.32 nM and 62.65 nM Compared to Control Group. Data are presented in mean ± SD. ** P <0.01; *** P <0.001

The q-PCR results show that the *miR-155-5p *expression increased significantly compared with the control group after the chitosan-combination miRNAs were transfected in the SKOV3, in terms of both 31,32 nm and 62,65 nm concentrations (P<0.05)(Figure 4). The miR-155-5p binding sites were in the HIF1α mRNA 3’UTR region as predicted. In order to verify that HIF1α was the target gene of the *miR-155-5p*, we measured the relative expression of *HIF1α*. The results showed that the *HIF1α* expression decreased significantly along with increased expression of endogenous miR-155-5p. This results showed than miR-155-5p could directly target HIF1α. Statistical analysis Pearson was measured to find out the correlation between the expression* miR-155-5p* and mRNA HIF1α. The results showed that p and r value respectively 0.000 and -0.930, indicated both of data have a strong negative correlations (P<0.05).

*MiR-324-5p expression and its correlation with GLI1*

**Figure 5:**
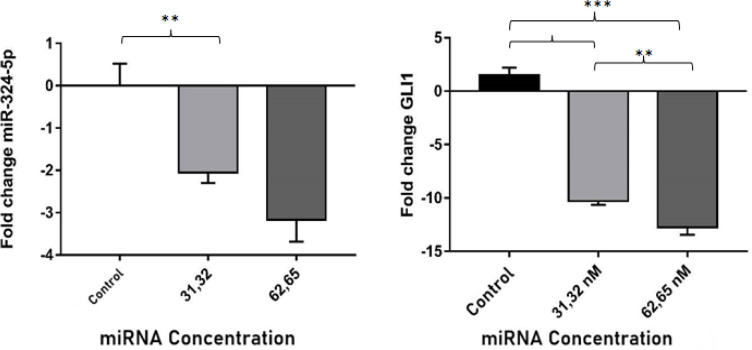
Fold change of endogenous miRNA-324-5p and mRNA GLI1 expression after transfected NPs-CS combination of miRNA 31.32 nM and 62.65 nM compared to the control group. Data are presented in mean ± SD. ** P <0.01; *** P <0.001

The q-PCR results show that the *miR-324-5p* expression decreased significantly compared with the control group after the chitosan-combination miRNAs were transfected in the SKOV3, in terms of both 31,32 nm and 62,65 nm concentrations (P<0.05)(Fig.5). The miR-324-5p binding sites were in the *GLI1 mRNA 3’UTR* region as predicted by Gene Card software. In order to verify that GLI1 was the target gene of the *miR-324-5p*, we measured the relative expression of *GLI1*. The results showed that the *GLI* expression decreased significantly along with decreased expression of endogenous* miR-324-5p*. This results showed than GLI1 could be targeted by miR-324-5p. Statistical analysis Spearman was measured to find out the correlation between the expression *miR-324-5p* and mRNA GLI1. The results showed that p value 0.020 (P<0.05) and Spearman value 0.750, indicated the expression of both have a positive correlations.

## Discussion

Chitosan nanoparticles is used as a carrier of microRNA due to a high positive charge and low toxicity. This result was due to the ability of nanoparticles to package the poly-anionic combination miR-155-5p and miR-324-5p by way of an electrostatic interaction which comes about between the negatively-charged phosphate residue of nucleic acid and the positively-charged amino-residues of chitosan. Chitosan nanoparticles formulation was carried out by ionic gelation method. The ionic gelation method involves a cross linker between the polyelectrolyte and its multivalent ion pair. The formation of a cross linker will strengthen of the particles formed. The polymer pair used for ionic gelation in this study was sodium tripolyphosphate. 

The ability of nanoparticles to preserve microRNA and the effectiveness of encapsulation was qualitatively measured using electrophoresis. The result showed that the formation of chitosan/TPP-microRNA has been marked by the absence of bands in the microRNA combination encapsulated chitosan. This result due to the size of encapsulated microRNA become heavier caused difficultness to run in the pores of the gel (Winarti et al., 2013). Morphological testing of chitosan nanoparticles-microRNA combinations in this study produced nanoparticles in spherical shape and did not overlapping. Spherical nanoparticles have a greater uptake than rod shapes (Chithrani et al., 2007; Nur Laili et al., 2014).

The 3-[4,5-dimethylthiazol-2-ly]-3,5-diphenyltetra-zolium bromide (MTT) test was performed on the SKOV3 cell lines in order to investigate the cytotoxicity of the colloidal formulation. MicroRNA as an anti-cancer candidate must be tested for its cytotoxicity to determine its inhibitory effect on SKOV3 cancer cells. The principle of this method is the addition of MTT reagents dissolved in PBS. Collection of enzymes found in mitochondria and cytosols of living cells, or known as mitochondrial dehydrogenase, will cut the tetrazole ring from MTT and reduce it to form purple formazan crystals.

Cytotoxicity tested with several variants of combinations miR-155-5p mimic and anti-miR-324-5p, both for naked miRNA and chitosan-miRNA nanoparticles. The dosage variations used were 50 nm, 60 nm, 70 nm, 80 nm, 90 nm and 100 nm. The result based on the graphic (Figure 3), variants of 100 nm concentration in miRNA nanoparticles can reduce cell viability ± 40%. This dose variation refers to the study of Xu et al., (2014) using mimic miR-324-5p which was transfected to U87 cells (human glioma) able to inhibit glioma cell proliferation by 60% with a variation of 100 nm dose. In other words, the higher the concentration of the miRNA combination used, the higher the average percentage of cell death.

MicroRNA works by attaching to its target mRNA and causing translational suppression or degradation of its target mRNA. Thus, the researchers also calculated the target mRNA expression level of *miR-155-5p*, which is HIF1α. Jiang et al., (2006) shows that HIF1α is strongly expressed in ovarian cancer. The high expression of *HIF1α *can increase the expression of both protein and *VEGF *mRNA, which results in tumor growth and angiogenesis. Many in silico studies using miRNA target prediction algorithms, such as miRanda, TargetScan, and PicTar state that miR-155-5p has proven to target HIF1α. Chasanah et al., (2016) showed that the expression of *miR-155-5p* in the blood serum of advanced stage ovarian cancer patients is lower than that of early-stage ovarian cancer patients. Correlates with increased *HIF1α* expression in patients with advanced stage ovarian cancer compared with patients with early-stage ovarian cancer.

The expression of endogenous* miR-155-5p* increased after being treated with miRNA combination nanoparticles caused decreased HIF1α expression in* SKOV3* ovarian cancer cells. Mimic miR-155-5p is designed to resemble endogenous miR-155-5p, so that it can be recognized by the RISC complex and can carry out the function of endogenous *miR-155-5p* in suppressing the expression of its target mRNA. Statistical tests also showed endogenous miR-155-5p negatively correlated with HIF1α, with a very strong correlation strength.

Hypoxia-inducible factor (HIF) is a transcription factor that regulates the expression of various genes to be able to adapt in hypoxic conditions. One of the key factors governing cellular hypoxia response is Hypoxia-inducible factor 1. HIF1 is a heterodimer consisting of HIF Subunits 1α and HIF-1β. HIF-1α activity is regulated by proteosomal degradation that is mediated everywhere under normoxia conditions. When cancer cells lack oxygen, HIF1α is stabilized and translocated to the nucleus, where HIF1α activates angiogenesis and anaerobic metabolism. This mechanism play a role in the survival of cancer cells. Decreased *HIF1α *expression can reduce the viability of SKOV3 cancer cells.

*MiR-324-5p* has a role as oncomiR which suppresses the expression of tumor suppressor genes. The expression *miRNA-324-5p* was decreased in the treated group compared to the control group. Chen et al., (2015) using anti-miR-21, which successfully suppresses the expression of endogenous miR-21 that is overexpressed. Anti-miR-21 blocks angiogenesis in breast cancer by inactivating the AKT and MAPK pathways.

According to Thomson et al., (2013) it is necessary to examine the expression of the target mRNA from miRNA to ensure that the transfected miRNA enters the target cell and performs its function to suppress gene expression. Results of *GLI1* expression analysis after the transfected of miRNA combination, decreased expression significantly compared to the control group. This fact is also supported by the results of correlation analysis between *miR-324-5p* expression and *GLI1* which has a positive correlation. Ciucci et al., (2013) study stated that the level of *GLI1 *expression was found to be significantly increased in ovarian cancer epithelial cells compared to normal ovarian tissue. Prove that the combination of miRNA encapsulated chitosan can reduce *GLI1* expression.

The molecular mechanism of miR-324-5p in targeting GLI1 in ovarian cancer cells is probably through Hedgehog pathway. Mir-324-5p may play an important role in key gene regulators in the Hedgehog pathway. Hedgehog (Hh) pathway is a signal transduction pathway which has the main function as regulating physiological processes. Hedgehog pathway has main components namely Hh ligand which consists of: Patched receptor protein (Ptch), Smoothened transmembrane protein (Smo), cytoplasmic protein (Fused kinase, Cos2, GSK3 beta, PKA, SuFu, GLI). GLI is a downstream regulator in Hedgehog signaling. GLI1 has a function as an activator of transcription factors. Hedgehog signaling is affected by binding of extracellular proteins to the Ptch receptor, Smo becomes overexpressed and causes activation of the GLI1 transcription factor. GLI1 induces several target genes that play a role in cell progression such as: Cyclin D1, c-Myc, and Bcl-2. Abnormal expression of *GLI1* affects the progression of cancer cells. The normal expression of *miR-155-5p* after tranfected can suppress the expression of *HIF1α*. The decreasing expression of *miR-324-5p *can suppress the expression of *GLI1*. This study successfully presented effects of combination of mimic miR-155-5p and antagonist miR-324-5p encapsulated chitosan which be considerated as a potential targets therapy for ovarium cancer.
